# Surface mineralized biphasic calcium phosphate ceramics loaded with urine-derived stem cells are effective in bone regeneration

**DOI:** 10.1186/s13018-019-1500-7

**Published:** 2019-12-09

**Authors:** Fei Xing, Lang Li, Jiachen Sun, Guoming Liu, Xin Duan, Jialei Chen, Ming Liu, Ye Long, Zhou Xiang

**Affiliations:** 1 Department of Orthopaedics, West China Hospital, Sichuan University, No. 37 Guoxue Lane, Chengdu, 610041 Sichuan People’s Republic of China; 2 Department of Pediatric Surgery, West China Hospital, Sichuan University, No. 37 Guoxue Lane, Chengdu, 610041 Sichuan People’s Republic of China

**Keywords:** Urine-derived stem cell, Biphasic calcium phosphate ceramics, Tissue engineering, Surface mineralization, Bone defects

## Abstract

**Background:**

Segmental bone defects caused by trauma, tumors, or infection are a serious challenge for orthopedists in the world. Recent developments in tissue engineering have provided a new treatment for segmental bone defects. Urine-derived stem cells (USCs) can be obtained noninvasively and might be a new kind of seed cells used in bone tissue regeneration. Therefore, the first aim of the present study was to investigate the biological characteristics of USCs. The second aim of the present study was to study the osteogenic effect of surface mineralized biphasic calcium phosphate ceramics (BCPs) loaded with USCs in vitro and in vivo.

**Methods:**

We isolated USCs from the urine of healthy adult donors and evaluated the biological characteristics of USCs in vitro. We mineralized the surface of BCPs by simulated body fluid (SBF). Cell adhesion and proliferation of USCs on the surface mineralized BCPs were evaluated. Osteogenic proteins and genes of USCs on the surface mineralized BCPs were texted by enzyme-linked immunosorbent assay (ELISA) and real-time polymerase chain reaction (RT-PCR) assay. Critical-sized segmental bone defects model in New Zealand white rabbits were established and randomly divided into 4 groups (surface mineralized BCPs loaded with USCs, BCPs loaded with USCs, surface mineralized BCPs, and BCPs) based on the implant they received. The therapeutic efficacy of the scaffolds in a large bone defect at post-implantation was evaluated by imaging and histological examination.

**Results:**

USCs isolated in our study expressed stem cell-specific phenotypes and had a stable proliferative capacity and multipotential differentiation capability. Surface mineralized BCPs promoted osteogenic proteins and genes expression of USCs without affecting the proliferation of USCs. After 10 weeks, the amount of new bone formation was the highest in the group of surface mineralized BCPs loaded with USCs.

**Conclusion:**

USCs, from non-invasive sources, have good application prospects in the field of bone tissue engineering. Surface mineralized BCPs can significantly enhance osteogenic potential of USCs without changing biological characteristics of BCPs. Surface mineralized BCPs loaded with USCs are effective in repairing of critical-sized segmental bone defects in rabbits.

## Background

Currently, bone marrow, adipose tissue, umbilical cord, umbilical cord blood, and placentas are the main sources of seed cells used in bone engineering [1]. However, the procedures to obtain stem cells from such sources are invasive. In 2008, Zhang et al. first reported the existence of urine-derived stem cells (USCs) [2]. Recently, many researchers have confirmed that USCs possess self-renewal ability and might be a new kind of seed cells used in bone regeneration [3–5]. As USCs can be obtained by noninvasive means, they represent a promising alternative kind of seed cells for bone regeneration [6]. At present, there are few studies focusing on the scaffolds loaded with USCs used in repair of segmental bone defects. It is not clear bone repair effect of scaffolds loaded with USCs in vivo.

As a kind of calcium phosphate (CaP), biphasic calcium phosphate ceramics (BCPs) have been widely used in the biomedical field because of its excellent biocompatibility and bioactivity [7]. BCPs consist of hydroxyapatite (HAp) and β-tricalcium phosphate (β-TCP), which are similar to the inorganic minerals of bone tissue [8]. The biological effects of BCPs, including protein adsorption, regulation of the osteogenic gene expression, biological degradation, biological risk, and anticancer activity, make it a preferred candidate for bone repair [9, 10]. Calcium ion released from BCPs can increase expression of growth factor, activate Ca sensing receptors in osteoblast cells, and enhance cell proliferation, differentiation, and mineralization [11]. In addition, compared to other kinds of biomaterials, BCPs are more beneficial to biomineralization in hard tissue regeneration [12]. The key to bone formation is bone calcification, also known as mineralization. The mineralization of biomaterials in vivo mainly refers to the formation of bone-like HAp, a biologically active apatite interface layer, on the surface of materials [13]. However, following the implantation of materials into bone defects, the process of surface mineralization takes a few weeks [14]. Therefore, we hypothesize that mineralization of materials in vitro, and combination with stem cells could promote and accelerate the process of bone repair. In addition, there is no research that has figured out whether apatite interface layer enhances osteoinductivity of BCPs on stem cells, especially USCs. Therefore, the first aim of our study is to investigate biological characteristics and multilineage differentiation potential of USCs. The second aim of our study is to evaluate the effect of surface mineralization of apatite interface layer on osteogenic potential of USCs in vitro and in vivo.

## Materials and methods

This study was approved by the institutional ethical review board of our institution. The Research Ethics Board for both human urine samples and animal protocols was approved by the Ethics Committee of West China Hospital, Sichuan University, Chengdu, China. Written informed consent was obtained from all participants. This study was performed under the Guide for the Care and Use of Laboratory Animals of the National Institutes of Health. This study was conducted in accordance with the Declaration of Helsinki.

### Isolation and culture of USCs

**Fig. 1:**
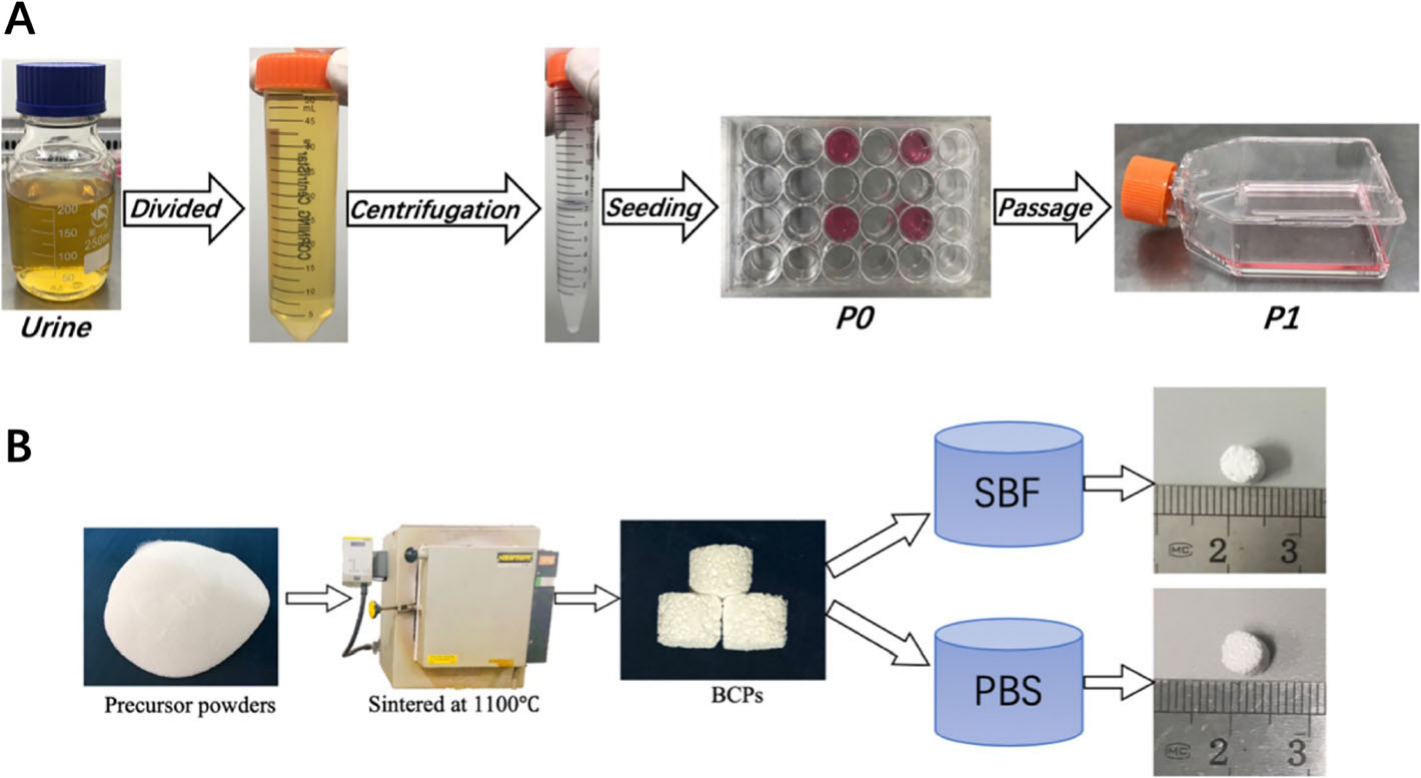
The isolation procedure of USCs and production process of BCPs of different groups

After obtaining informed consent, we collected 200–250 mL urine samples from healthy adult male donors (age range 25–35 years) and transferred them to 50-mL centrifuge tubes. Each urine sample was centrifuged at 200×*g* for 10 min and the supernatant was discarded. We resuspended and washed the pellets twice with phosphate-buffered saline (PBS). The cell pellets were then resuspended in embryo fibroblast medium (EFM) and keratinocyte serum-free medium (KSFM) (1:1 ratio), and seeded into 24-well culture plates. We cultured the cells in a 5% CO_2_ humidified cell incubator at 37 °C. Single-cell clones appeared on the plates after about 7 days. When the monolayer of cells covered more than 80% of the bottom of the plate, cells were trypsinized and passaged into cell culture dishes for expansion. We used cells at the fourth passage for our experiment. A brief summary of USC isolation procedure is provided in Fig. 1a.

### Biological characteristics of USCs

#### Cell proliferation

To generate a growth curve, we seeded USCs onto 96-well plates at a density of 4500 cells/well and measured cell proliferation on days 1–10 using Cell Counting Kit-8 (CCK8, APExBIO Technology LLC, USA). Briefly, cells were incubated with 100 μL of fresh medium containing 10 μL of CCK8 solution in a humidified incubator for 1 h at 37 °C. After incubation, we transferred the supernatant to a new 96-well plate and measured the optical density (OD) at 450 nm using a spectrophotometer.

#### Identification of cell phenotype

The suspension of USCs was washed twice with PBS and stained with the following specific antihuman antibodies: CD29, CD44, CD90, CD31, CD45, and HLA-DR. Further information on antibodies is provided in Table 1. Briefly, USCs was trypsinized and resuspended in PBS containing 1% bovine serum albumin. Fluorochrome-conjugated antibodies were added to PBS suspension of USCs, and the suspension was incubated on ice for 0.5 h in the dark. We used conjugated isotype control antibodies to subtract the background fluorescence. Finally, the suspension of USCs was analyzed by flow cytometry using FlowJo software.

**Table 1:** Antibodies information of USCs used in this study

Antibody	Souce/Cat No.	Isotype
CD90-FITC	BD Biosciences/No.555595	Mouse BALB/c IgG_1_, κ
CD29-PE	BD Biosciences/No.557332	Mouse BALB/c IgG_1_, κ
CD44-FITC	BD Biosciences/No.555478	Mouse IgG_2b_, κ
CD31-FITC	BD Biosciences/No.557508	Mouse IgG_1_, κ
CD45-FITC	BD Biosciences/No.555482	Mouse IgG_1_, κ
HLA-DR-FITC	BD Biosciences/No.560944	Mouse IgG_2a_, κ

#### Multilineage differentiation

To evaluate osteogenic differentiation of USCs, we seeded 2 × 10^4^ cells/well into a 6-well plate. When the cells covered 80–90% of the bottom of the plate, we replaced the medium with 2.5 mL of human MSC osteogenic differentiation medium. Osteogenic medium was Dulbecco’s modified eagle medium supplemented with 10% fetal bovine serum, 50 μg/mL ascorbic acid, 0.2 μM dexamethasone, and 10 mM of β glycerol phosphate disodium. We changed the osteogenic differentiation medium every 3 days. After 28 days, we removed the medium from wells, rinsed with PBS, and fixed cells with 2 mL of 4% paraformaldehyde solution for 30 min. After fixation, we rinsed wells with PBS and stained the cells with 1 mL Alizarin Red S working solution for 10 min. Finally, we rinsed wells twice with PBS and observed the staining results under a microscope. To assess the adipogenic differentiation of USCs, we seeded the USCs into a 6-well plate at a density of 8 × 10^4^ cells/well and replaced the medium with 2.5 mL of human MSC adipogenic differentiation medium. The adipogenic medium was DMEM HG plus 10% FBS, 1 μM dexamethasone, 10 μg/mL bovine insulin, 50 μM indomethacin, and 500 μM isobutylmethylxanthine. We cultured the USCs in adipogenic induction medium. After 21 days, we removed the medium from wells, rinsed with PBS, and fixed cells with 2 mL of 4% paraformaldehyde solution for 30 min. After fixation, we rinsed wells with PBS and stained the cells with 1 mL Oil Red O working solution for 10 min. Finally, we rinsed wells twice with PBS and observed the staining results under a microscope. To evaluate chondrogenic differentiation, we seeded 10^4^ cells/well into a 6-well plate, and replaced the medium with 2.5 mL of human MSC chondrogenic differentiation medium. Chondrogenic medium was DMEM LG supplemented with 50 μg/mL L-ascorbic acid, 5 ng/mL human recombinant TGF-β3, and 6 μg/mL of human insulin. We cultured the USCs in chondrogenic differentiation medium. After 21 days, we removed the medium from wells, rinsed with PBS, and fixed cells with 2 mL of 75% ethanol for 20 min. After fixation, we rinsed wells with PBS and stained the cells with 1 mL Alcian blue working solution for 30 min. Finally, we rinsed wells twice with PBS and observed the staining results under a microscope.

### Material preparation

The BCPs comprised HA and β-TCP in a 30/70 ratio. The BCPs were fabricated by H_2_O_2_ foaming according to a method described in the literature [15]. Briefly, we mixed 1.0 M Ca(NO_3_)_2_ with 1.0 M (NH_4_)_2_HPO_4_ according to a specified Ca/P ratio, and we maintained a pH of 10.0 by mixing with NH_4_OH. After washing with deionized water and spray-drying, the mixed slurries broke into powders. After adding 10% polyvinyl alcohol, 30% H_2_O_2_, and 3% methyl cellulose to the precursor powders, we stirred the resulting slurries, dried them in a mold, sintered them at 1100 °C, and cut them into cylinders (φ5 × 5 mm).

The composition of the SBF is presented in Table 2. The SBF had the same ionic composition as human plasma and was produced in accordance with a method described in the literature [16]. Briefly, we prepared the fluid by dissolving NaCl, NaHCO_3_, Na_2_CO_3_, KCl, K_2_HPO_4_, MgCl_2_·6H_2_O, CaCl_2_, and Na_2_SO_4_ in that order; 4-(2-hydroxyethyl)-1-piperazineethanesulfonic acid and NaOH were used to maintain the pH value at 7.4. This SBF solution can be stored at 4 °C in a refrigerator. We then placed the BCPs in a 24-well plate with 2 mL of SBF solution and incubated the plate at 37.0 °C in an oven. The 24-well plate solution was changed every day. After 14 days, we removed the BCPs from the SBF, rinsed them with PBS, dried them in an oven, and sterilized then for in vitro culture. A schematic diagram of material preparation is shown in Fig. 1b.

**Table 2:** The composition of the SBF used in this study

Ion	SBF (molm^−3^)	Plasma (molm^−3^)
Mg^2+^	1.5	1.5
Ca^2+^	2.5	2.5
K^+^	5.0	5.0
Na^+^	142.0	142.0
HCO_3_^−^	4.2	27
HPO_4_^2−^	1.0	1.0
SO_4_^2−^	0.5	0.5
Cl^−^	147.8	103.0

### Characteristics of scaffolds

Scanning electron microscopy (SEM) and X-ray diffraction (XRD) were used to evaluate the results of surface mineralization. As a control, we incubated the BCPs in PBS for 14 days. The porosity of the groups of BCPs and surface mineralized BCPs were measured using the Mercury intrusion method. The mechanical compression tests of BCPs and surface mineralized BCPs were carried out by mechanical universal tension machine, and the displacement rate was set to 1 mm/min. The samples were compressed until the 10% strain. A tangent modulus was fit to the linear portion of the stress-strain curve using Matlab. The linear portion was defined as the last 2% strain of the ramping phase of the 10% increment.

### Cell seeding

For cell seeding, 6 × 10^5^ USCs at the fourth passage were directly seeded onto scaffolds. For in vitro experiments, USCs were allowed to adhere onto the surface of scaffolds for 4 h before the addition of culture media. For in vivo experiments, the USCs/scaffolds composites were pre-cultured in USCs in the osteogenic differentiation media for 7 days.

### Viability and proliferation of USCs on scaffolds

Calcein AM and propidium iodide (PI) stock solutions were diluted into working solutions with final concentrations of 2 μmol/L and 4 μmol/L, respectively. The working solutions were added to scaffolds and incubated in the dark for 10 min at 37 °C. We evaluated the results by confocal laser-scanning microscopy. Live cells attached to the scaffolds were stained green with Calcein AM, and dead cells were stained red with PI. The cytoskeleton of USCs on the surface of scaffolds was evaluated by rhodamine phalloidin staining. After culturing USCs on the scaffolds for 2 days, USCs were permeabilized for 10 min with 0.5% Triton X-100. The scaffolds were incubated in rhodamine phalloidin solution in the dark for 25 min at 37.0 °C, and in 4′,6-diamidino-2-phenylindole (DAPI) solution in the dark for 5 min at room temperature. We used a confocal laser-scanning microscope to capture the images.

We evaluated the proliferation of USCs on the scaffolds using a CCK8 assay. After culture, we examined the USCs on days 1, 3, and 7. At each time-point, the USCs and scaffolds were incubated with the CCK8 working solution in a humidified incubator with 5% CO_2_ at 37 °C for 2 h. Finally, we measured the absorbance of the soaking solution at 450 nm using a full-wavelength microplate reader.

### Osteogenic proteins secreted by USCs attached to scaffolds

We collected the cell culture supernate on days 1, 3, 7, and 14, and stored them at −20 °C. The osteogenic proteins secreted by the attached USCs, including alkaline phosphatase (ALP, Signalway Antibody, USA) and osteocalcin (OCN, Signalway Antibody, USA), were analyzed by enzyme-linked immunosorbent assay (ELISA). Briefly, ELISA is based on a competitive binding enzyme immunoassay technique. Standards or samples are then added to the appropriate microtiter plate wells, which have been pre-coated with a specific antibody. We added avidin-conjugated horseradish peroxidase to each well and incubated for 30 min, then added a 3,3′,5,5′-tetramethylbenzidine substrate solution to each well. The enzyme-substrate reaction was terminated by adding sulfuric acid solution, and the color change was measured at a wavelength of 450 nm using a spectrophotometer. We determined the osteogenic protein concentrations of the samples by comparing their ODs to those of the standard curve.

### Osteogenic genes expression of USCs on scaffolds

To investigate osteogenic genes expression of USCs in scaffolds, we carried out quantitative reverse transcription-polymerase chain reaction (qRT-PCR) assays at days 3, 7, and 14. We evaluated the expression levels of several specific osteogenic markers including early markers such as alkaline phosphatase (ALP), bone morphogenetic protein-2 (BMP2), and runt-related transcription factor-2 (RUNX2), and late markers such as osteocalcin (OCN).

The attached USCs were cultured in osteoblast-inducing conditional media (Life Technologies, Gibco), and total RNA was extracted with an RNA extraction kit according to the manufacturer’s instructions on days 3, 7, and 14. We used GAPDH as a housekeeping gene during the analysis. Total RNA was extracted with TRIzol solution (Invitrogen, Carlsbad, CA, USA), and was reverse transcribed into cDNA using a QuantiTect Reverse Transcription Kit (Thermo Fisher Scientific Inc, Fremont, CA, USA) according to the manufacturer’s instructions. We amplified the specific transcripts using a SYBR Green Mix Kit (Roche Company, Berlin, Germany) on a real-time fluorescence quantitative instrument. We used the primer sequences listed in Table 3 for qRT-PCR analysis. PCR was performed over 40 cycles, each comprising 94 °C for 15 s, 55 °C for 30 s, and 72 °C for 30 s. Marker gene expression data were analyzed by the 2-ΔΔCT method using GAPDH. All PCRs were carried out in triplicate.

**Table 3:** Primers for real-time polymerase chain reaction

Target gene	Forward primer sequence (5-3)	Reverse primer sequence (5-3)
RUNX2	CCAACCCACGAATGCACTATC	TAGTGAGTGGTGGCGGACATAC
BMP2	ACCCGCTGTCTTCTAGCGT	TTTCAGGCCGAACATGCTGAG
ALP	ACCACCACGAGAGTGAACCA	CGTTGTCTGAGTACCAGTCCC
OCN	CCCCCTCTAGCCTAGGACC	ACCAGGTAATGCCAGTTTGC
GAPDH	ACAACTTTGGTATCGTGGAAGG	GCCATCACGCCACAGTTTC

### Establishment of critical-sized segmental defects model in vivo

**Fig. 2:**
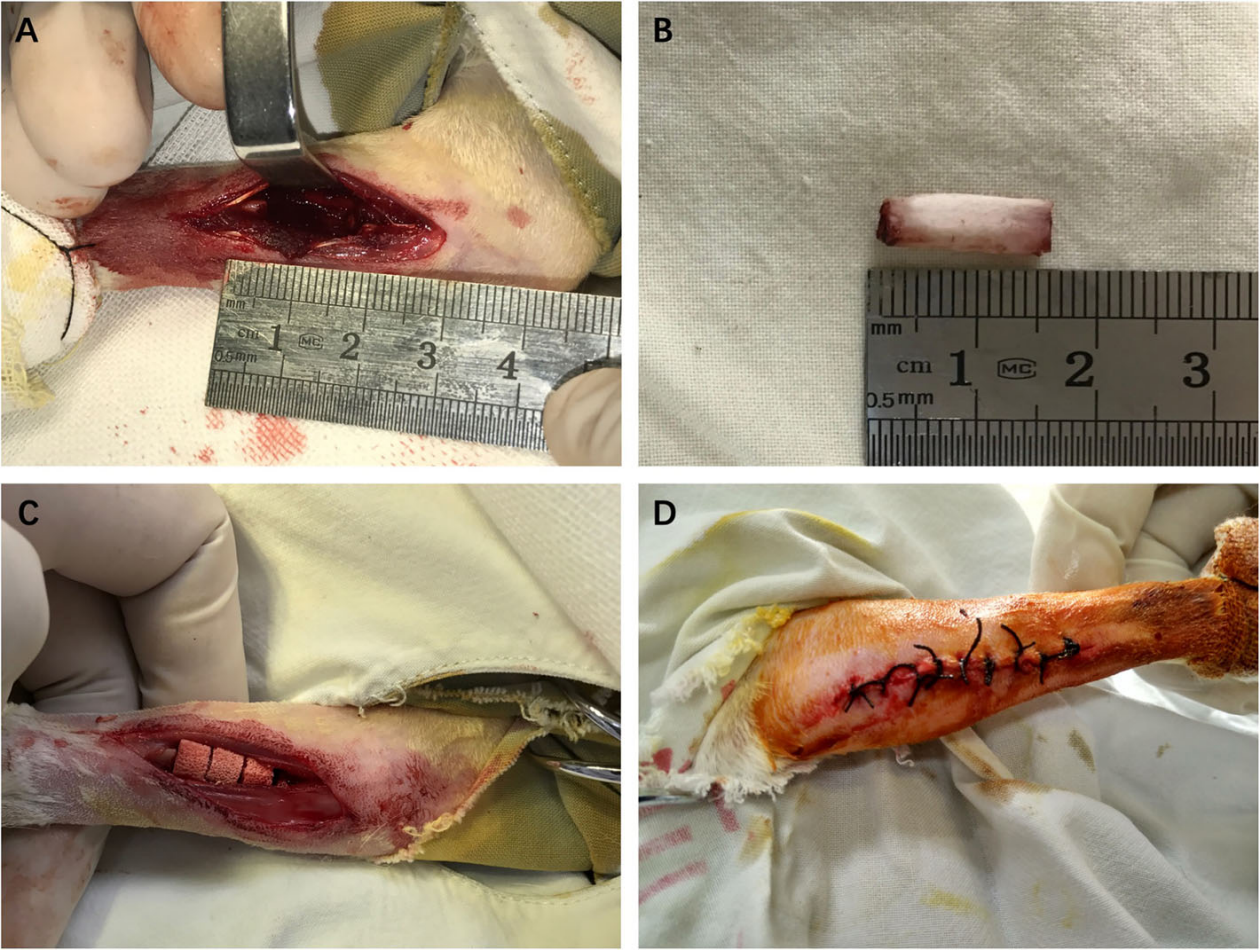
Establishment of critical-sized segmental bone defects model in vivo. **a** A longitudinal incision along the middle of the forelimb of about 3 cm in length was made. **b** A 15 mm length of ulna bone was removed. **c** Scaffolds loaded with cells were implanted in the defect area; **d** Wound closure

Adult healthy New Zealand white rabbits weighing 2.5–3.0 kg were selected for establishing a model of critical-sized segmental defects. After anesthesia, a longitudinal incision along the forelimb of about 3 cm in length was made by the surgeon, followed by incision of the skin and subcutaneous tissues in layers. The surgeon exposed the ulna via the intermuscular space, removed 15 mm length of ulna bone together with the periosteum. After implanting different groups of materials, the wounds were carefully flushed with saline and closed layer by layer. The critical-sized segmental defects model was performed in both sides of the rabbit’s forelimb. The establishment of the model of critical-sized segmental defects in New Zealand white rabbits is shown in Fig. 2. Twelve animals were randomly divided into 4 groups (surface mineralized BCPs loaded with USCs, BCPs loaded with USCs, surface mineralized BCPs, and BCPs) based on the implant they received.

### Assessment of repair effect of critical-sized segmental bone defects

After 10 weeks, the rabbits were sacrificed by an overdose of anesthesia. Then, ulnas from each group were assessed using Micro-CT. The relative structural integrity of bone bridging into the ulna defect was evaluated by the three-point bending test. We fixed the two ends of the ulna on the mechanical tester to make the longitudinal axis of ulna parallel to the ground and performed three-point bending test. The loading site was a bone defect repair area. The test was set at a start point of 0.1 N load, a loading speed of 2 mm/min, and a stop point of 0.85 mm flexural extension. For histological assessment, the samples were fixed in 10% neutral formalin for 5–7 days and decalcified with 10% EDTA decalcification solution for 4 weeks. Routine gradient dehydration, xylene transparency, paraffin embedding, section, haematoxylin and eosin (HE) staining and Masson staining were performed.

### Statistical analysis

All data are reported as the means ± standard deviation (SD). We used one-way analysis of variance (ANOVA) for statistical analysis of the differences between the study groups. We took *p* values of < 0.05 as an indication of a statistically significant difference. SPSS Statistics version 22.0 (IBM Corp, Armonk, NY, USA) was used to perform all the statistical analyses.

## Results

### Biological characteristics of USCs

**Fig. 3:**
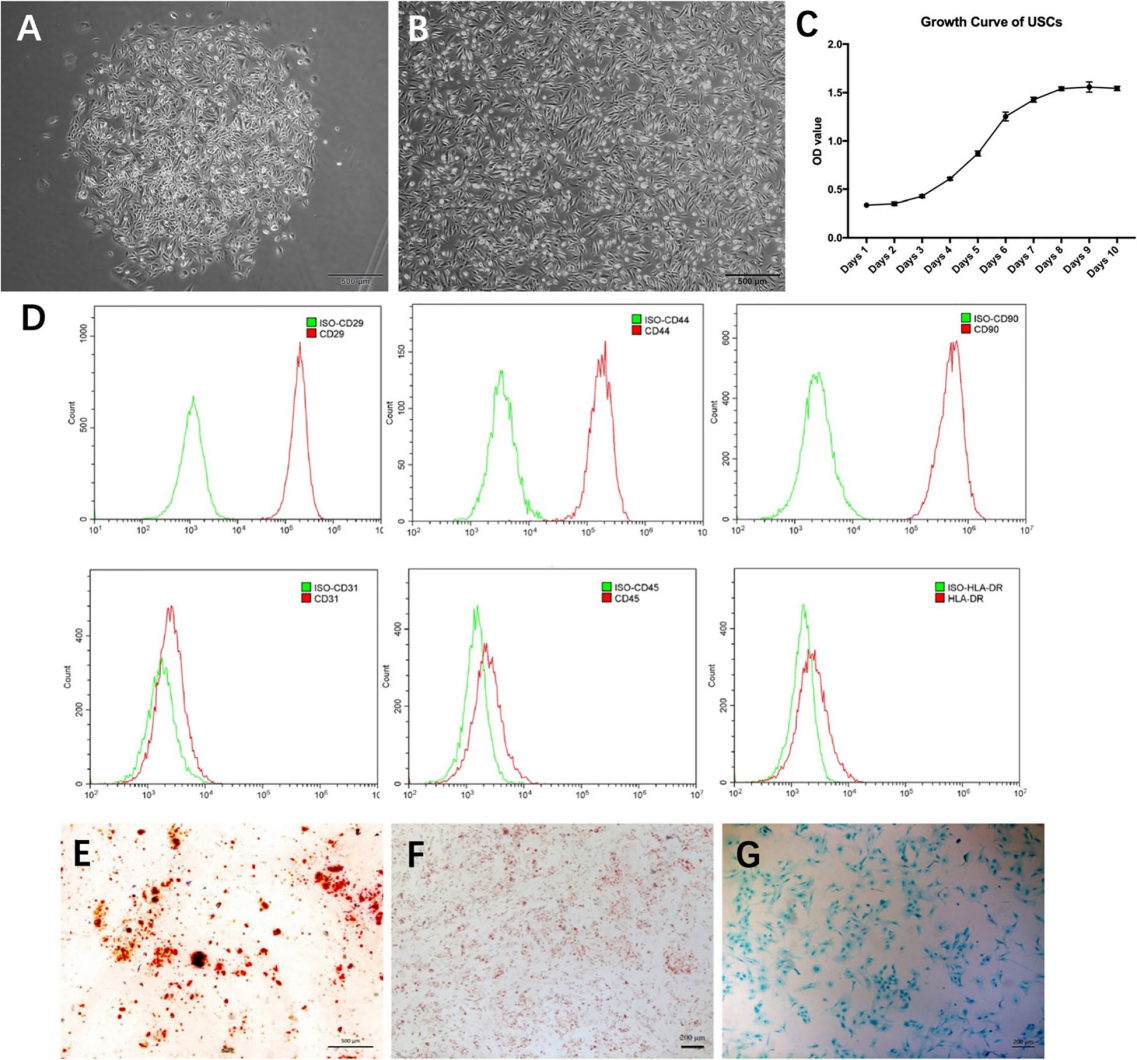
Biological characterization of USCs. **a**–**b** The morphology of USCs at different passages. **c** The growth curve of USCs **d** Flow cytometric analysis demonstrates that urine stem cells were positive for CD29, CD44, CD90, and negative for CD45, CD31, HLA-DR. **e** Osteogenic differentiation was demonstrated by Alizarin red S staining. **f** Adipogenic differentiation was demonstrated by Oil Red O staining. **g** Chondrogenic differentiation was demonstrated by toluidine blue staining

The typical cell colonies were observed at 8–10 days after culture (Fig. 3a). The isolated USCs had a morphology resembling rice grains. After several passages, the isolated USCs elongated and exhibited fibroblast-like morphology (Fig. 3b). To generate a growth curve, we seeded USCs onto 96-well plates at a density of 4500 cells/well. The growth curve of USCs was typically S-shaped (Fig. 3c). Flow cytometric analysis revealed that the USCs were positive for MSC markers CD29, CD44, and CD90; and negative for endothelial lineage marker CD31, hematopoietic lineage marker CD45, and immunocyte marker HLA-DR (Fig. 3d). The USCs were cultured in specific induction media, which were used to determine the trilineage differentiation capabilities of the USCs. After osteogenic differentiation, we identified calcium nodules by Alizarin Red staining (Fig. 3e). Oil Red O staining revealed lipid vacuoles after the adipogenic differentiation of the USCs (Fig. 3e). Toluidine blue staining indicated the chondrogenic differentiation capability of the USCs (Fig. 3g).

### Characteristics of scaffolds

**Fig. 4:**
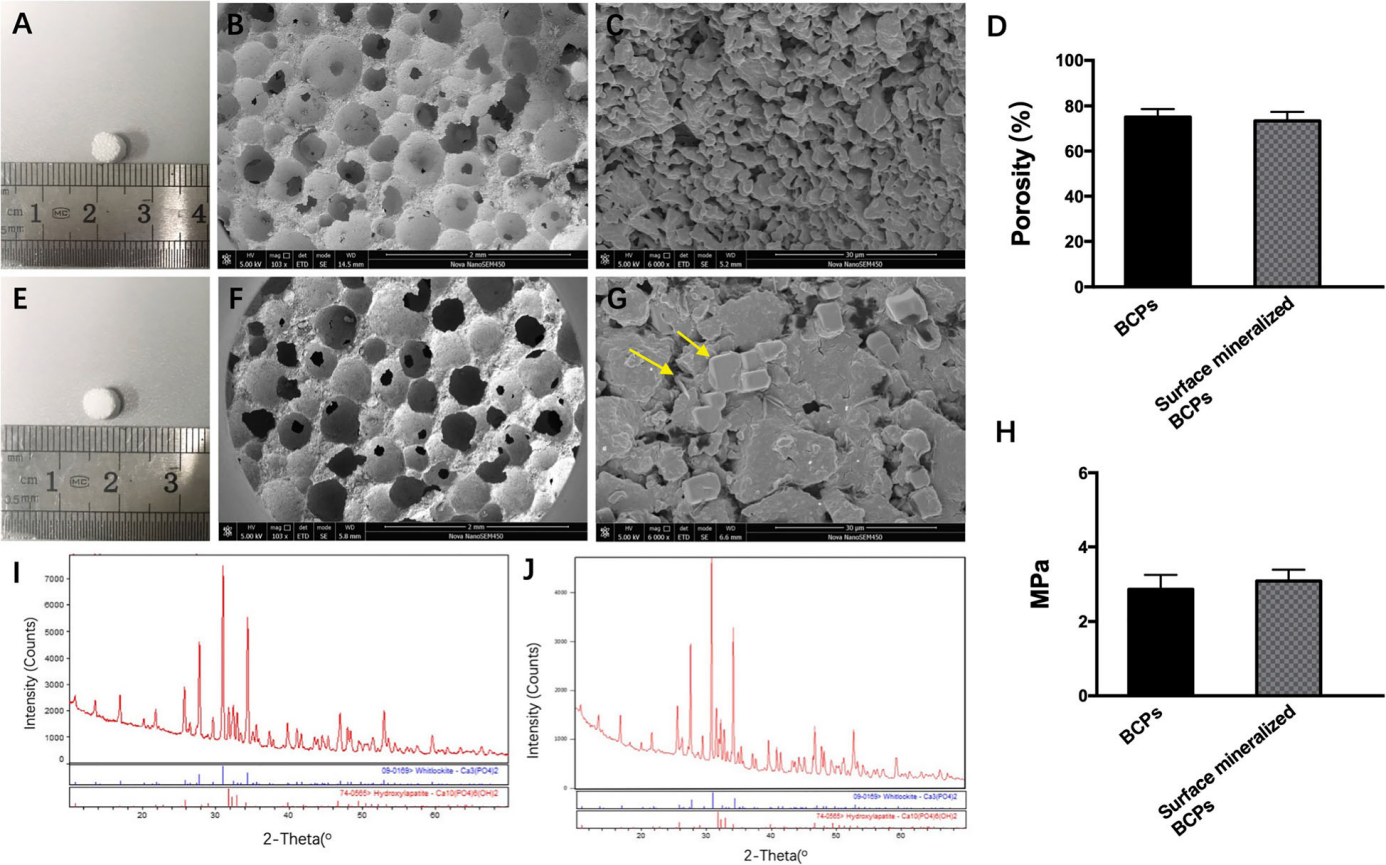
SEM and XRD of BCPs and surface mineralized BCPs. **a**–**c** General and SEM observation of BCPs. **d** The porosity of BCPs and surface mineralized BCPs. **e–g** General and SEM observation of surface mineralized and BCPs. **h** Mechanical compression testing. **i–j** XRD of BCPs and surface mineralized and BCPs. *P < 0.05, and those with no symbol show no significant difference.

We assessed the surface morphologies and microstructures of the various groups of scaffolds by general observation and SEM. General observation showed that both groups of scaffolds are white and porous. (Fig. 4a, e) After immersion in SBF solution for several days, plate and sheet crystals (yellow arrows) were formed on the surface of BCPs. (Fig. 4f, g) The results of XRD of two groups show no significant difference, which indicated that plate and sheet crystals on the surface of BCPs comprised bone-like HAp. (Fig. 4i, j)

The average porosity of BCPs was 75.02 ± 3.64%, while the average porosity of surface mineralized BCPs was 73.37 ± 4.01%. There was no significant difference between the two groups. (Fig. 4d) The pore size of BCPs and surface mineralized BCPs ranged from 200 μm to 400 μm. The pores were interconnected by micropores. In addition, mechanical compression testing showed no significance between the groups of BCPs and surface mineralized BCPs. (Fig. 4h)

### Viability and proliferation of USCs on scaffolds

**Fig. 5:**
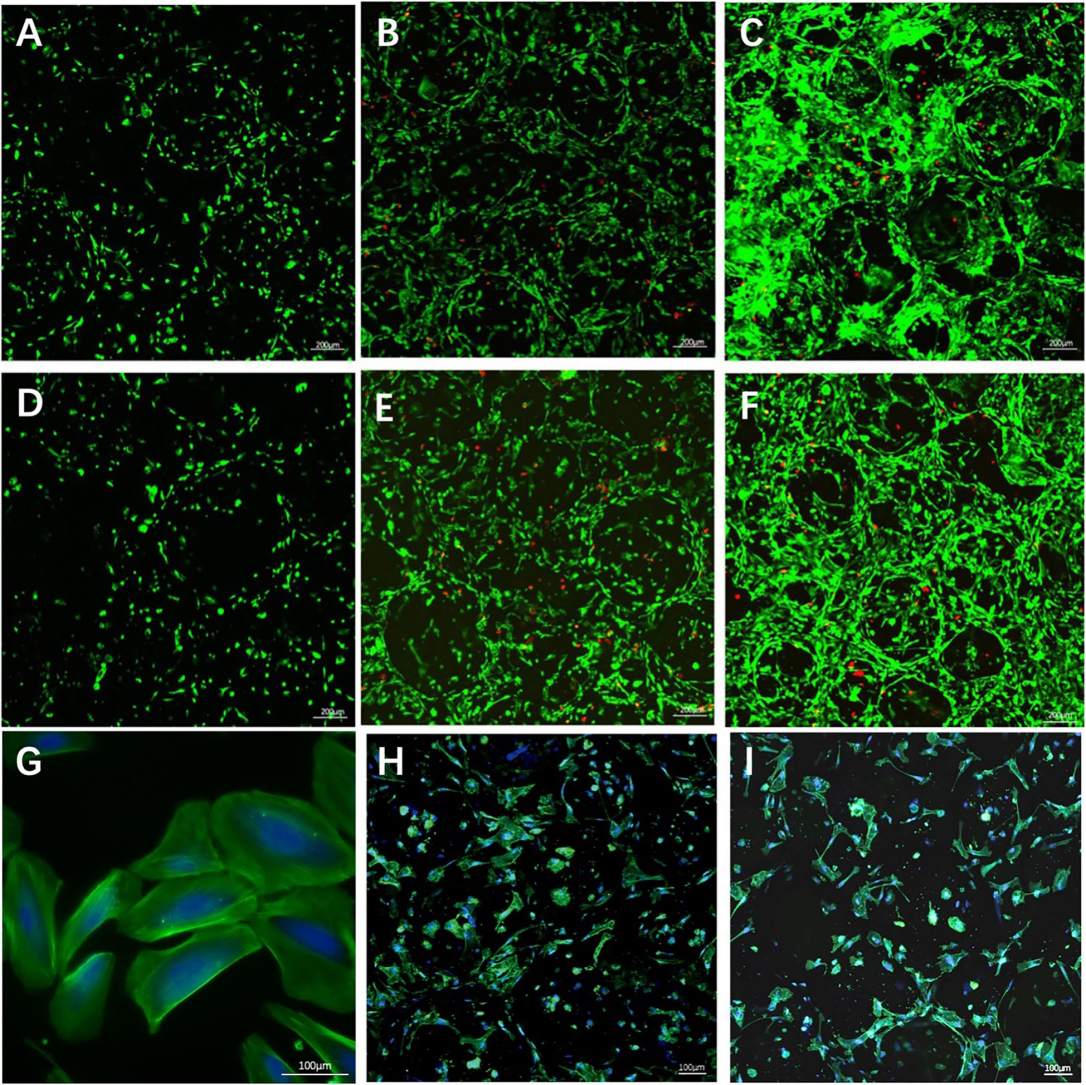
Calcein AM/PI staining and rhodamine phalloidin staining of USCs on BCPs and surface mineralized BCPs. **a–c** Calcein AM/PI staining of USCs cultured on BCPs on days 1, 3, and 7. **d–f** Calcein AM/PI staining of USCs cultured on surface mineralized BCPs on days 1, 3, and 7. **g** Cytoskeleton of USCs on the surface of cell cultures. **h** Cytoskeleton of USCs on the surface of BCPs. **i** Cytoskeleton of USCs on the surface of surface mineralized BCPs

**Fig. 6:**
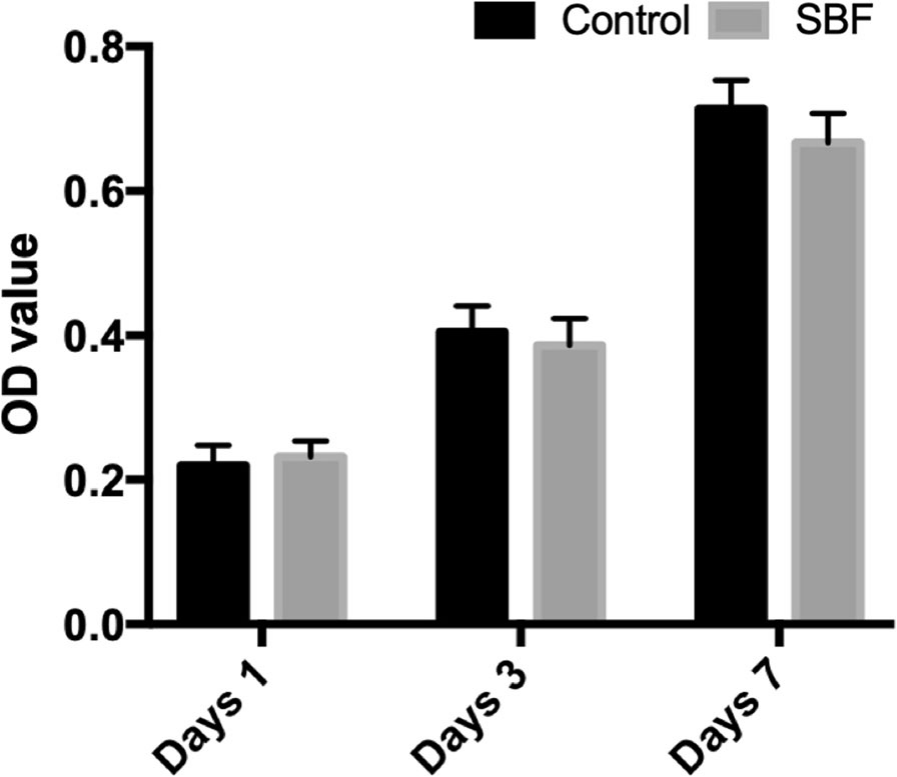
The proliferation of USCs on BCPs and surface mineralized BCPs

We evaluated the viability of the USCs cultured on the various scaffolds by seeding 6 × 10^5^ USCs onto each of various groups of scaffolds. Figure 5 showed that the densities of the USCs on the surfaces of the various groups of scaffolds increased with culture time. The CCK8 results showed no significant difference between the groups of surface mineralized BCPs and BCPs on days 1 and 3, and 7 days, which indicated, compared with BCPs, surface mineralized BCPs had no effect on proliferation of USCs (Fig. 6). Figure 5g, i illustrates the results of rhodamine phalloidin/DAPI staining in the actin filaments of USCs. The isolated USCs had rice grain-like morphologies. Furthermore, after seeded onto the surface of scaffolds in the two groups, USCs exhibited good adhesion and became polygonal.

### Osteogenic protein secreted by USCs on scaffolds

**Fig. 7:**
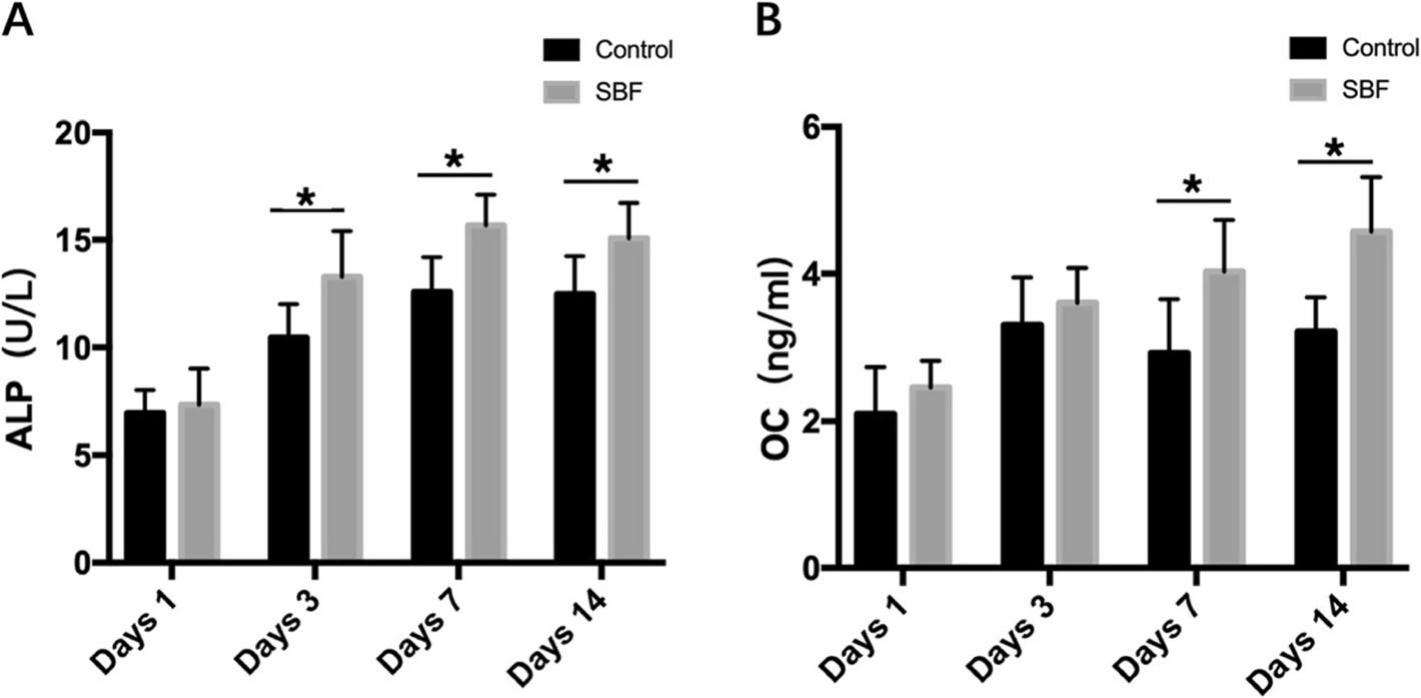
Osteogenic protein secretion by attached USCs in different groups of BCPs. **a** ALP secreted on days 1, 3, 7, and 14. **b** OCN secreted on days 1, 3, 7, and 14. *P < 0.05, and those with no symbol show no significant difference

After seeding 6 × 10^5^ USCs onto scaffolds, we evaluated the expression levels of osteogenic protein in the culture supernate, on days 1, 3, 7, and 14 after osteogenic induction. The results showed that the levels of osteogenic proteins, including ALP and OCN, increased when USCs were seeded onto scaffolds (Fig. 7). OCN is a vital protein in the regulation of bone metabolism. The OCN of group of surface mineralized BCPs loaded with USCs (4.03 ± 0.70 ng/mL) on day 7 was significantly higher than group of BCPs loaded with USCs (2.92 ± 0.73 ng/mL). On day 14, the OCN of the group of surface mineralized BCPs loaded with USCs (4.57 ± 0.75 ng/mL) was significantly also higher than the group of BCPs loaded with USCs (3.21 ± 0.47 ng/mL). The ALP content of group of surface mineralized BCPs loaded with USCs on day 3, day 7, day 14 was 13.28 ± 2.13 U/L, 15.68 ± 1.43 U/L, and 15.08 ± 1.64 U/L, respectively, which were significantly higher than group of BCPs loaded with USCs (10.46 ± 1.55 U/L, 12.58 ± 1.61 U/L, 12.48 ± 1.77 U/L).

### Expression of osteogenic genes of USCs in scaffolds

**Fig. 8:**
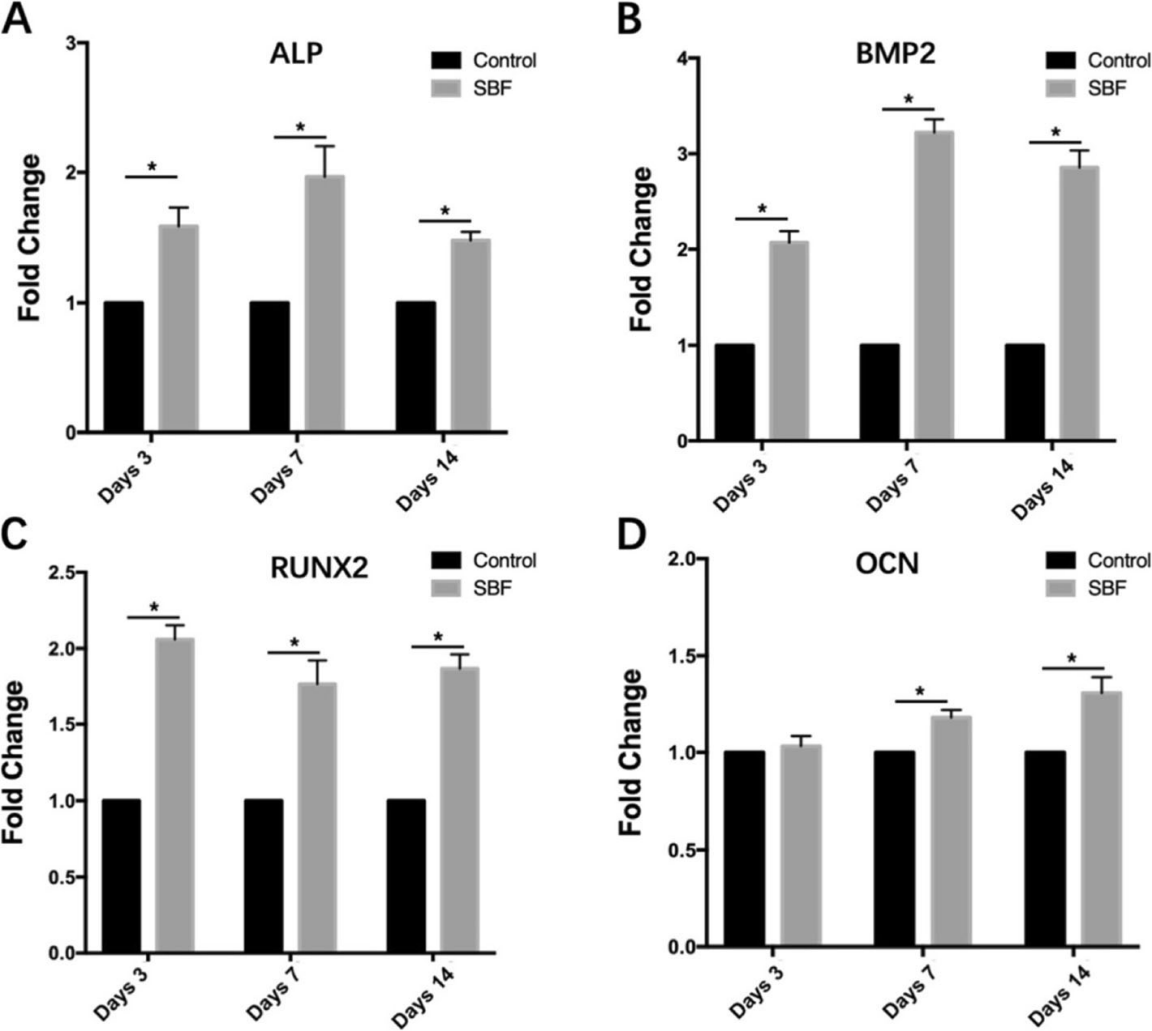
RT-PCR analysis for the gene expression of USCs on BCPs treated with PBS, SBF on days 3, 7, and 14 (*n* = 3). The mRNA expression was normalized to GAPDH. The mRNA levels shown are relative to the mRNA level of control group. **a** Gene expression of ALP. **b** Gene expression of RUNX2. **c** Gene expression of BMP2. **d** Gene expression of OCN. *P < 0.05, and those with no symbol show no significant difference

We used qRT-PCR analysis to measure the mRNA expression levels of osteogenic genes, including ALP, RUNX2, BMP2, and OCN, at various time-points by seeding 6 × 10^5^ USCs onto scaffolds. Compared to the group of BCPs loaded with USCs, the gene expression levels of ALP, RUNX2, BMP2, and OCN in the group of surface mineralized BCPs loaded with USCs were significantly higher on days 7 and 14 (Fig. 8). On day 3, the expression levels of ALP, BMP2, and RUNX2 in the group of surface mineralized BCPs loaded with USCs were significantly higher than those in the group of BCPs loaded with USCs. There was no obvious difference in the level of OCN expression between the two groups on day 3.

### Assessment of repair of critical-sized segmental bone defects

**Fig. 9:**
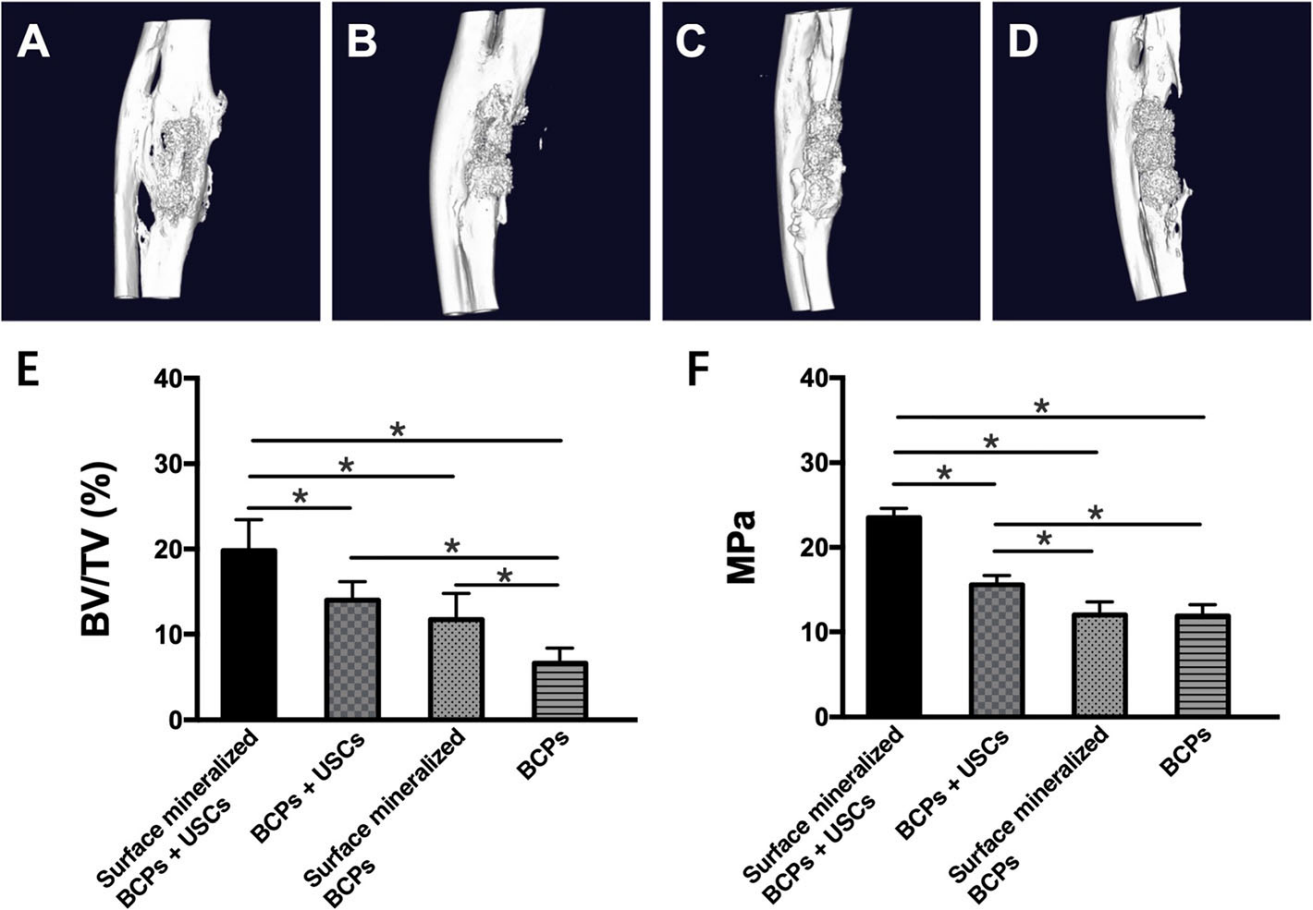
Micro-CT Assessment of repair of critical-sized segmental bone defects. **a** Surface mineralized BCPs loaded with USCs. **b** BCPs loaded with USCs. **c** Surface mineralized BCPs. **d** BCPs. **e** Quantification of the newly formed bone in the implant. BV/TV refers to bone volume/total volume. **f** Three-point bending test results. *P < 0.05, and those with no symbol show no significant difference

For in vivo experiments, we seeded 6 × 10^5^ USCs at the fourth passage onto scaffolds and pre-cultured in the osteogenic differentiation media for 7 days. We evaluated the bone repair ability of different groups in animal mode of critical-sized segmental bone defects, which is 15 mm bone defect of ulna in rabbits [17]. The results of Micro-CT showed the degradation of scaffolds and the formation of new bone were observed in all four groups after 10 weeks. Except for the group of BCPs, callus formation was observed in the junction of scaffolds and bone in the other three groups. In addition, bone volume/total volume (BV/TV) was calculated and used for quantitative analysis (Fig. 9). The BV/TV was as follows: 19.82 ± 3.65, 13.97 ± 2.23, 11.72 ± 3.08, and 6.65 ± 1.78 for groups of surface mineralized BCPs + USCs, BCPs + USCs, surface mineralized BCPs, and BCPs, respectively. The BV/TV of group of surface mineralized BCPs + USCs was significantly higher than the other three groups. In addition, the BV/TV of group of BCPs was lowest among the four groups. There was no significant difference between groups of BCPs + USCs and surface mineralized BCPs.

Three-point bending test was used to evaluate the bone structural stiffness. Our data showed that the bone bridging of the group of surface mineralized BCPs + USCs was significantly stiffer than the other three groups. In addition, the bone bridging of the group of BCPs + USCs was significantly stiffer than that of the group of BCPs. No significant difference was observed between the groups of surface mineralized BCPs and BCPs (Fig. 9f)

**Fig. 10:**
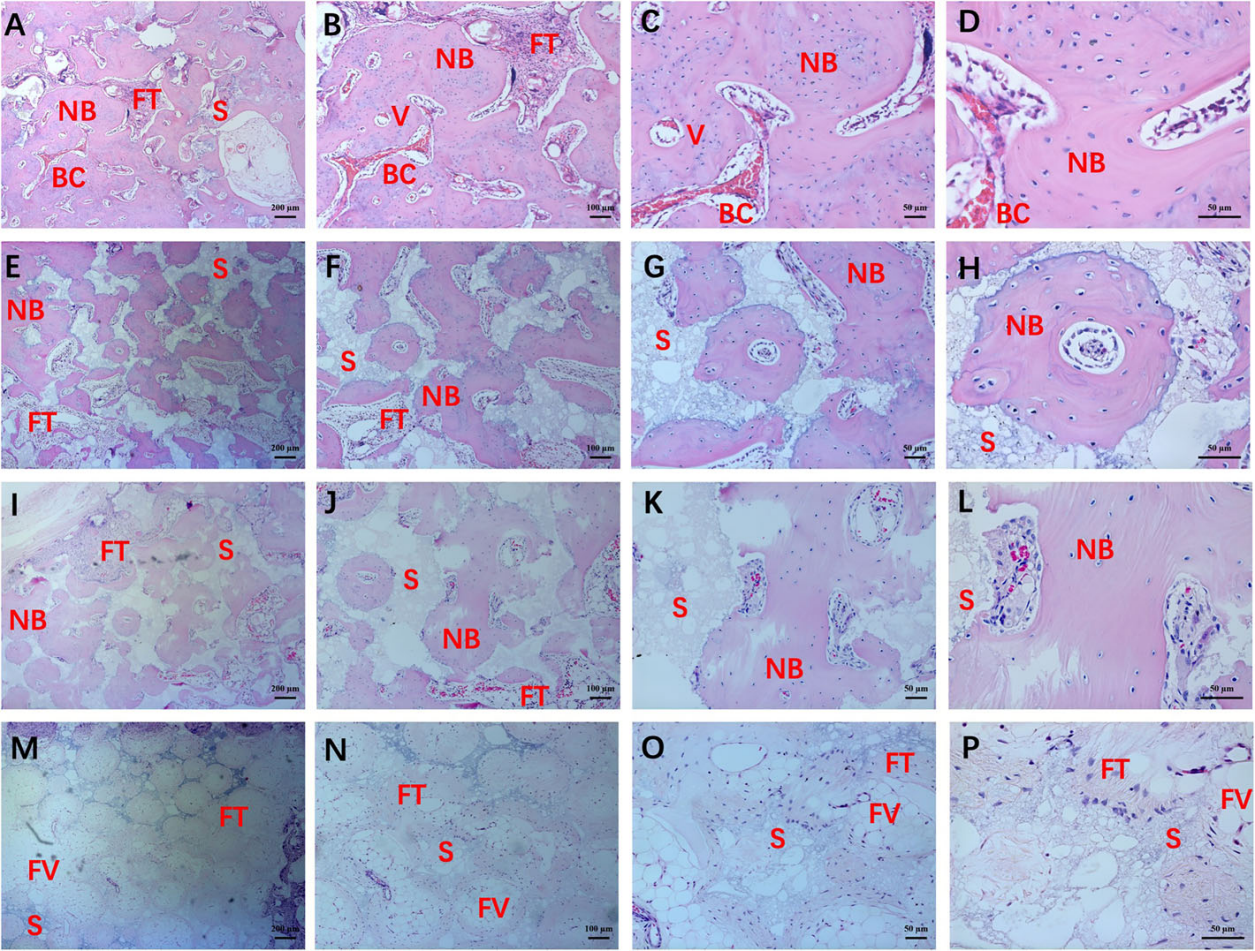
The HE staining of bone defects after post-implantation at different multiples. **a**–**d** HE staining of group of surface mineralized BCPs loaded with USCs. **e–h** HE staining of group of BCPs loaded with USCs. **i–l** HE staining of group of surface mineralized BCPs. (M-P) HE staining of group of BCPs. *NB*: new bone; *BC*: blood cell; *FT*: fibrous tissue; *S*: scaffolds; *V*: vessel; *FV*: fat vacuoles

HE staining and Masson staining were used to evaluate the therapeutic efficacy of the scaffolds in critical-sized segmental bone defects at post-implantation. The results of HE staining of four groups were shown in Fig. 10. The group of BCPs showed massive fibrous tissue and fat vacuoles in the defect and did not form numerous new bones. In the group of surface mineralized BCPs and BCPs loaded with USCs, new bone formation and a small amount of fibrous tissue were was observed. In addition, an amount of undegraded scaffolds was also observed in groups of surface mineralized BCPs and BCPs loaded with USCs. Compared to the above three groups, the group of surface mineralized BCPs loaded with USCs had more new bone formation. In addition, only a small amount of undegraded scaffolds was observed. Interestingly, some blood cells and vessels were also found in the group of surface mineralized BCPs loaded with USCs.

**Fig. 11:**
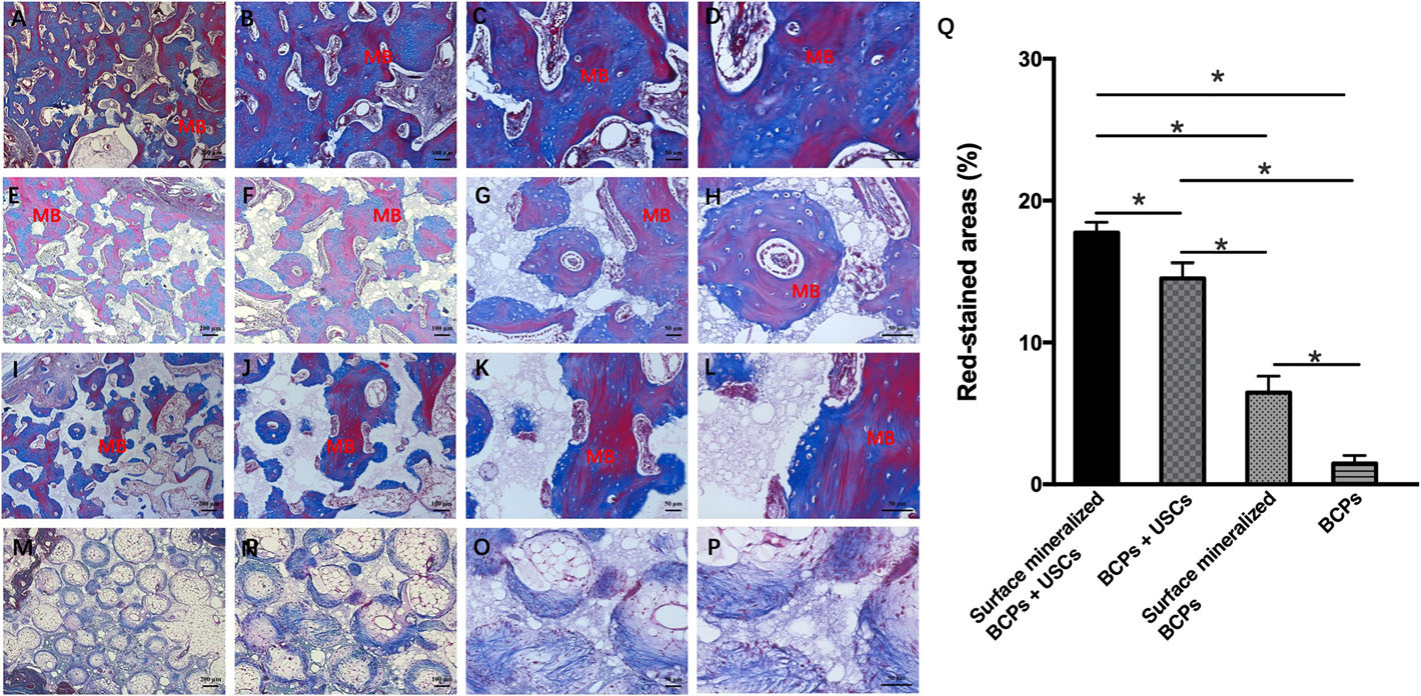
The Masson staining of bone defects after post-implantation at different multiples. **a–d** Masson staining of group of surface mineralized BCPs loaded with USCs. **e-h** Masson staining of group of BCPs loaded with USCs. **i–l** Masson staining of group of surface mineralized BCPs. **m–p** Masson staining of group of BCPs. **q** Semiquantitative results of red-stained areas based on Masson staining of four groups. *MB*: mature bone. *P < 0.05, and those with no symbol show no significant difference

Masson’s trichrome staining was also used to evaluate the bone formation in vivo (Fig. 11). Abundant fat vacuoles were observed in the group of BCPs, and the result was in accordance with that from H&E staining. Except for the group of BCPs, a large number of bone lacunas were observed in the other three groups. In addition, the new bone in the group of surface mineralized BCPs loaded with USCs was dense and continuous, while the new bone in groups of BCPs loaded with USC and surface mineralized BCPs was dispersed and discontinuous. In addition, the red-dyed areas were mature bone matrix, which indicated the maturity of the collagen fibers in bone defects. The semiquantitative results (Fig. 11q) of the Masson staining revealed that the average red-stained areas in the group of surface mineralized BCPs loaded with USCs were significantly higher than the other three groups, which indicated the maturity of the bone matrix was higher in the group of surface mineralized BCPs loaded with USCs.

## Discussion

Our study confirmed that USCs expressed MSC markers, had good cell proliferation ability and multiple differentiation potential which was consistent with the results of previous studies [18]. In addition, previous studies have found differences in the biological characteristics of USCs from children, adult, and elderly donors [19]. In order to reduce the biological differences between USCs from different donors, the USCs used in this study were all from healthy male donors aged between 25 and 35 years old. In addition, USCs were obtained by specially trained laboratory technicians through the same separation process. In addition, the medium used in our study has specific selectivity for USCs [18]. After selection and passage, USCs with stable cell morphology at the fourth passage were used in our study. In addition, previous studies found that USCs can impart profound immunomodulatory effects, by inhibiting proliferation of peripheral blood mononuclear cells (PBMNC) and T and B cells, and secreting interleukin (IL)-6 and IL-8 [19]. PBMNCs proliferated, when mixed with other cells due to immune stimulation [20]. PBMNC concentrations in USC wells were much lower than in BMSC culture wells [19]. However, there are still few studies focusing on the immunoregulatory property of USCs. More studies are needed to verify the immunoregulatory property of USCs.

Similar to connective tissue from other sources, bone tissue consists of cells, fibers, and matrix. However, the strongest feature of bone is that there is a large amount of calcium deposition in the interstitium. Calcium salts are relatively insoluble, especially phosphate and carbonate. However, apatite crystals are soluble and widely participate in circulation of extracellular fluid and bone formation. The key to bone formation is mineralization. The mineralization process involves the conversion of amorphous phosphate into crystalline form [13]. However, following the implantation of materials into bone defects, the process of surface mineralization takes a few weeks [14]. Therefore, mineralization of the scaffold surface in vitro before implantation might be helpful to promote and shorten the process of bone repair. In addition, previous study found that surface mineralization before application ensures that the scaffolds anchor to the bone more easily, and improves the bone-bonding capability [20, 21]. SBF comprises a solution with ion concentrations close to those found in human blood plasma, and has often been used to simulate the inner environment of the human body [22]. In addition, SBF has been widely used to promote biomaterials to generate a mineralized layer in vitro, which could strengthen cellular osteogenic activities, improve mineral deposition, and promote bone remodeling [23, 24]. Compared with other forms of coating, surface mineralization by SBF treatment mimics natural crystallization process [25]. Our study found that SBF can promote the formation of plate and sheet apatite crystals on the surface of scaffolds. Compared with control group, no new substances were detected by XRD in the group of scaffolds immersed in SBF, which indicated that the plate and sheet crystals on the surface of the scaffolds were composed of bone-like HAp. Our results found that surface mineralized BCPs had no effect on viability and proliferation of attached USCs compared with BCPs, which indicated that surface mineralized BCPs could also provide a favorable microenvironment that enables USCs to adhere to scaffolds, survive, proliferate. In addition, our results demonstrated that the surface mineralization layer had no effect on the porosity of BCPs.

Currently, many present researchers focus on enhancing the osteogenic potential of USCs by gene transfection and other methods [3, 26]. However, there are few studies focusing on enhancing the osteogenic capacity of USCs on scaffolds by changing the properties of scaffolds. Therefore, our study focused on enhancing the osteogenic potential of USCs from three-dimensional perspective by mineralizing scaffolds without affecting the biological characteristics of scaffolds. Our study found that after soaking in SBF, flake apatite crystals (surface mineralization layer) formed on the surface of the BCPs. Surface mineralization of BCPs involves dissolution, deposition, and the growth of a new solid phase from the liquid phase [27]. In our study, SBF provided the BCPs with a suitable supersaturated environment, which facilitated cluster nucleation and crystal calcium phosphate formation. In addition, previous studies found that a biologically active hydroxycarbonate apatite layer can mediate bone marrow MSCs to condensate and spontaneously differentiate toward osteogenic lineage [28]. ALP is a homodimeric protein enzyme that plays a vital role in the metabolism of osteoblast activity, which increases if there is active bone formation [29]. Osteocalcin (OCN) is a biochemical marker for bone formation. OCN is secreted from osteoblasts and plays a vital role in bone-building [30]. In our study, compare with BCPs, surface mineralized BCPs enhance the ALP and OCN expression of USCs. Osteogenic gene, included early osteogenic gene markers (ALP, BMP2, and RUNX2) and late osteogenic gene marker (OCN), were continuously and significantly upregulated in the group of surface mineralized BCPs loaded with USCs. The results of gene and protein expression showed that surface mineralization enhanced the osteogenic potential of USCs in vitro.

Quantification of the static parameters of bone formation by micro-CT analysis reveals that the BV/TV is significantly higher in the group of surface mineralized BCPs loaded with USCs, compared to those of other groups. Histological results show that group of surface mineralized BCPs loaded with USCs had the best bone formation ability and the fastest rate of scaffolds degradation compared with the other three groups. Compared to the other three groups, more mature bone matrix was observed in the group of surface mineralized BCPs loaded with USCs, which indicated that the presence of USCs and surface mineralization layer promoted the formation of new bone and accelerated the maturation of new bone in the ulna defects.

Bone structural stiffness is another important factor in the degree of bone bridging and maturity [31]. In our study, the three-point bending test was used to evaluate the bone structural stiffness on the defect margins. Compared to groups of scaffolds without USCs (BCPs and surface mineralized BCPs), the groups of scaffolds loaded with USCs are significantly stiffer, which indicated that USCs could effectively improve the stiffness of materials in vivo. In addition, no significant differences were observed in stiffness between groups of BCPs and surface mineralized BCPs in vivo and in vitro*,* which indicated the surface mineralization layer has no effect on the stiffness of materials.

There are several limitations to our study. First, although this study analyzed the composition and formation mechanism of the mineralized layer, we did not explore the specific signaling pathway of the mineralized layer promoting the osteogenic potential of USCs. Second, USCs might face a high risk of bacterial infection, and the culture procedure is time-costing. In our study, we isolated USCs from midstream urine to decrease the risk of bacterial infection. In addition, antibiotics are added into urine before the process of centrifugation. In our opinions, the microcarrier culture technique might be a potential method to shorten the culture procedure of USCs. Third, although the immunoregulatory property of USCs was demonstrated, the USCs isolated from human beings were used in New Zealand white rabbits, which might induce immunoreaction. In our opinions, tracer techniques of stem cells might be a potential method to observe the immune microenvironment modulation in vivo. Finally, the observation time point of animal experiment section was single. It was not explored in depth whether the USCs were directly differentiated into osteocytes for repair or they were treated by stimulating the production of related cytokines

## Conclusion

To conclude, USCs isolated in our study expressed stem cell-specific phenotypes and had stable proliferative capacity and multipotential differentiation capability. USCs are non-invasive and have broad application prospects in tissue engineering. Surface mineralized BCPs can significantly enhance the osteogenic potential USCs in vitro without changing the biological characteristic of BCPs. Surface mineralized BCPs loaded with USCs were found to be effective in repairing of critical-sized segmental bone defects in rabbits.

## Data Availability

The datasets used and/or analysed during the current study are available from the corresponding author on reasonable request.

## Abbreviations

BCPs: Biphasic calcium phosphate ceramics
EFM: Embryo fibroblast medium
ELISA: Enzyme-linked immunosorbent assay
KSFM: Keratinocyte serum-free medium
MSCs: Mesenchymal stem cells
PBS: Phosphate-buffered saline
qRT-PCR: Quantitative reverse transcription polymerase chain reaction
SBF: Simulated body fluid; USCs: Urine-derived stem cells
